# Integrated analysis of long noncoding RNAs and mRNAs reveals their potential roles in the pathogenesis of uterine leiomyomas

**DOI:** 10.18632/oncotarget.2349

**Published:** 2014-08-16

**Authors:** Haiyang Guo, Xiyu Zhang, Ruifen Dong, Xiaolin Liu, Yinuo Li, Shan Lu, Limei Xu, Yuqiong Wang, Xiyao Wang, Dong Hou, Jian-Jun Wei, Changshun Shao, Zhaojian Liu

**Affiliations:** ^1^ Ministry of Education Key Laboratory of Experimental Teratology and Department of Molecular Medicine and Genetics; ^2^ Department of Cell Biology, Shandong University School of Medicine, Shandong University, Jinan, Shandong, China; ^3^ Department of Obstetrics and Gynecology, Qilu Hospital, Shandong University, Jinan, Shandong, China; ^4^ Department of Pathology, Northwestern University School of Medicine, Chicago, IL,USA

**Keywords:** lncRNA, protein coding genes, microarray, uterine leiomyoma,CAR Intergenic 10

## Abstract

Global expression profiling studies showed that miRNAs are aberrantly expressed in uterine leiomyomas (ULMs) and are involved in ULM pathogenesis. Long noncoding RNAs (lncRNAs) are another group of regulatory RNA whose expression and roles in ULMs have not been explored. In this study, we examined the global expressions of lncRNAs and mRNAs in ULMs using microarray and interrogated their interrelationship through co-expression analysis. We found that lncRNAs and mRNAs were dysregulated in ULMs and the degree of dysregulation was positively correlated with tumor size. Further analysis showed that lncRNAs correlate to their cis mRNAs in expression levels depending on genomic locations and orientations. Moreover, we identified several dysregulated pathways that were correlated to dysregulated lncRNAs. We validated several aberrantly expressed lncRNAs in extended samples and confirmed that *AK023096* was down-regulated and chromatin-associated RNA (CAR) *Intergenic 10* was up-regulated in the majority of leiomyomas. Knockdown of *Intergenic 10* inhibited the proliferation of leiomyoma cells in vitro, indicating its functional importance in ULM pathogenesis. The neighboring protein-coding gene *ADAM12* was also downregulated in *Intergenic 10* knockdown leiomyoma cells. We showed for the first time that lncRNAs were dysregulated in uterine leiomyomas. Aberrantly expressed lncRNAs may contribute to the pathogenesis of uterine leiomyomas.

## INTRODUCTION

Uterine leiomyomas (ULMs) are the most common benign smooth muscle tumors in women of reproductive age. These benign tumors originate from uterine myometrial cells and can cause uterine bleeding, pelvic pain and infertility[1]. The molecular mechanism underlying uterine leiomyoma tumorigenesis and development is not fully understood. Global gene expression analysis of ULMs revealed that hundreds of genes were dysregulated that are involved in cell proliferation, differentiation and extracellular matrix production [2, 3]. Epigenetic alterations may also contribute to the formation and progression of ULMs[4]. Recent studies showed that a subset of miRNAs was also abnormally expressed in ULMs and several miRNAs may play an important role in the tumorigenesis of uterine leiomyomas[5-7].

Long noncoding RNAs (lncRNAs) are transcripts of greater than 200 nucleotides without evident protein coding function[8]. LncRNAs encompass different classes of RNA transcripts, including enhancer RNAs, small nucleolar RNA (snoRNA) hosts, intergenic transcripts, and transcripts overlapping other transcripts in either sense or antisense orientation[9]. LncRNAs may function as guides, scaffolds, and decoys and thus have the potential to regulate gene expression and spatial localization within the cell[9]. LncRNAs have been shown to be involved in chromosome dosage-compensation, imprinting, epigenetic regulation, cell cycle control, nuclear and cytoplasmic trafficking, transcription, translation, splicing, cell differentiation[10]. Accumulating evidence suggests that aberrant expression of lncRNAs plays an important role in carcinogenesis. For example, lncRNA HOTAIR is overexpressed in breast cancer and overexpression of HOTAIR has been shown to drive breast cancer metastasis in vivo[9]. LncRNAs can act both as tumor suppressor genes (GAS5, LincRNA-p21 and PTENP1) and oncogenes (HOTAIR, MALAT1)[11-15].

Given the genetic complexity of human uterine leiomyomas, it is possible that aberrantly expressed lncRNAs may contribute to tumorigenesis of uterine leiomyomas. Therefore, in this study, we performed microarray-based genome-wide analysis of lncRNAs and mRNAs in leiomyoma and matched myometrium. We then developed a correlation matrix based integrative analysis approach to analyze lncRNA and mRNA co-expression data. Selected mRNAs and lncRNAs were validated by qPCR. We further performed functional analysis of chromatin-associated RNA (CAR) *Intergenic 10*, one of the up-regulated lncRNAs, to determine its potential role in ULMs development. Our findings suggest that lncRNAs may play an important role in pathogenesis of uterine leiomyoma.

## RESULTS

### Expression patterns of lncRNAs and mRNAs in uterine leiomyomas and matched myometrium

**Fig. 1:**
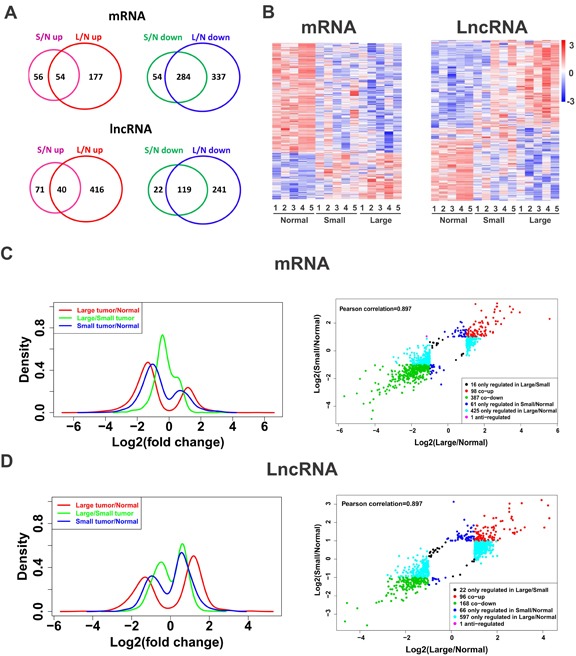
Altered global expression patterns of lncRNA and mRNA in leiomyomas (A) Venn diagrams showing up-regulated and down-regulated mRNAs and lncRNAs in leiomyomas (L, large; S, small; N, normal). (B) Hierarchically clustered heatmaps of 988 mRNAs and 949 lncRNAs that are altered (fold change > 2, p < 0.05). (C-D) The log2 (fold change) distribution patterns of differentially expressed mRNAs (C) and lncRNAs (D) (fold change > 2, p < 0.05). The density plots show the distributions of log2 fold change. The scatter plots show the distribution of log2 fold change of L/N matching of S/N.

Global expressions of lncRNAs and mRNAs in ULMs and matched myometrium were examined by Arraystar Human LncRNA Microarray v2.0 (Arraystar, Rockville, MD). Those lncRNAs and mRNAs showing 2 fold change and reaching statistical significance (P<0.05) between ULMs and matched myometrium were selected for further analysis. We found that 252 lncRNAs and 448 mRNAs were significantly dysregulated in small leiomyomas (<=3cm) compared with normal tissues. In contrast, 816 lncRNAs and 792 mRNAs were significantly dysregulated in large leiomyomas (Figure 1A). These data suggest that both LncRNAs and mRNAs are dysregulated in ULMs and the degree of dysregulation in gene expression was positively correlated with tumor size.

We then performed unsupervised hierarchical cluster analysis of differential expression of lncRNAs and mRNAs in uterine leiomyomas. As shown in Figure 1B, lncRNAs and mRNAs showed distinct expression profiles. Consistent with previous studies [16], the down-regulated mRNAs predominate among the dysregulated mRNAs. In contrast, up-regulated lncRNAs account for the majority of the dysregulated lncRNAs in ULMs (Figure 1B). Accordingly, the peak is higher in the down-regulated area than that in the up-regulated area in the density plots of log2 fold changes of mRNAs (Figure 1C), whereas the higher peak falls in the up-regulated area for lncRNA (Figure 1D). Thus, in general, mRNAs and lncRNAs were dysregulated in opposite directions. Density plots also revealed that dysregulated mRNAs and lncRNAs had a wider distribution in large tumors than in small tumors (Figure 1C and 1D). These data, together with those shown in Venn diagram (Figure 1A), indicate that more lncRNAs and mRNAs become dysregulated in large tumors than in small tumors, which implies that the large tumors acquire more genetic alterations than the small ones.

### Expression patterns of different lncRNA subtypes and their correlation with protein-coding genes

**Fig. 2:**
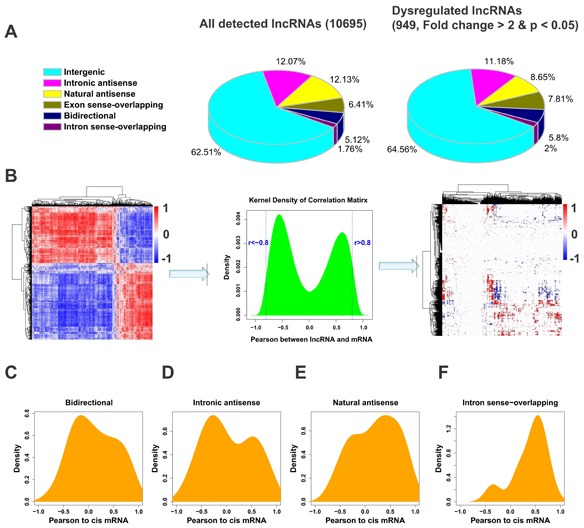
Correlation between the expression patterns of lncRNAs and their cis mRNAs (A) The fraction of all detected lncRNAs and dysregulated lncRNAs. The lncRNAs were classified as six subgroups (intergenic, intronic antisence, natural antisense, exon sense-overlapping, bidirectional and intron sense-overlapping) according to their relationships with their associated protein-coding genes. (B) Construction of correlation matrix. Left: A hierarchically clustered heatmap of the correlation between 988 mRNAs and 949 lncRNAs altered (fold change > 2, p < 0.05) in small or large tumors. Middle: The cumulative distributions of correlation coefficients. Values that are smaller than -0.8 are assigned as negative correlation and those greater than 0.8 as positive correlation and the others no correlation. Right: Correlation matrix after cut off. Red color indicates positive correlation, blue color indicates negative correlation and white represents no correlation. (C-F) The distributions of Pearson correlations between lncRNAs and their cis mRNAs were separately analyzed in four of the six lncRNA subgroups.

LncRNAs can be divided into different subtypes according to their genomic context in association with protein-coding genes, with the intergenic lncRNA subgroup accounting for the majority of the lncRNAs. As expected, the majority of the dysregulated lncRNAs belong to the category of intergenic lncRNA (Figure 2A). Other subgroups also appeared to be proportionally dysregulated in ULMs. Although several studies suggest that the majority of lncRNAs may function as trans-regulators [17, 18], the spatiotemporal relation between lncRNAs expression and their nearby protein-coding genes (cis mRNA) remains controversial [19-21]. To determine whether the lncRNAs and their cis mRNAs are correlated in expression, we calculated pairwise Pearson correlations in expression levels between lncRNAs (excluding intergenic and exon sense-overlapping lncRNAs) and their cis mRNAs by density plot (Figure 2B). Interestingly, each of the four subgroups of lncRNAs showed a unique distribution pattern in relationship to the expression levels of their cis mRNAs (Figure 2C-2F). Intronic antisense subgroup showed a bimodal distribution, though with a larger peak of negative correlation (Figure 2D). The bidirectional and natural antisense lncRNAs appeared to have opposite effects on the cis mRNA in general (Figure 2C and 2E). Intron sense-overlapping subtype showed an overwhelmingly positive correlation with their cis mRNAs (Figure 2F). These results suggest that lncRNAs may regulate the expression of their cis mRNAs differently depending on their genomic location and orientation to the related mRNAs.

### Identification of mRNA and lncRNA signatures in uterine leiomyomas

**Fig. 3:**
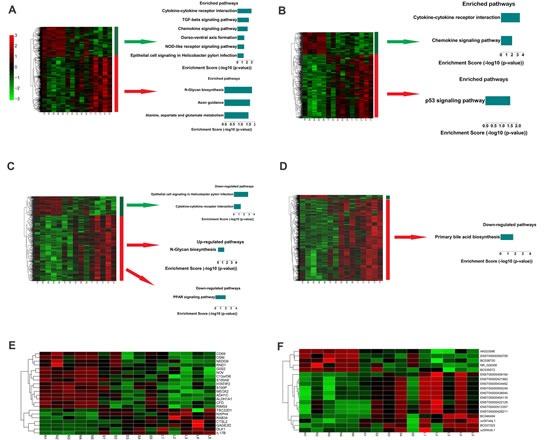
Signatures of altered mRNA and lncRNA expression in large leiomyomas (A) Left: A hierarchically clustered heatmap of mRNAs changed (fold change > 2) in large fibroids but not in small fibroids. Right: Significantly enriched pathways of the indicated gene sets. (B) Left: A hierarchically clustered heatmap of mRNAs that were more dramatically changed in large fibroids than in small fibroids. Right: Significantly enriched pathways of the indicated gene sets. (C) Left: A hierarchically clustered heatmap of lncRNAs changed (fold change > 2) in large fibroids but not in small fibroids. Right: Significantly enriched pathways of genes correlated with the indicated lncRNA sets. (D) Left: A hierarchically clustered heatmap of lncRNAs that are more dramatically changed in large fibroids than in small fibroids. Right: Significantly enriched pathways of genes correlated with the indicated lncRNA sets. (E) Significantly changed mRNAs (fold change (L/S) > 2). (F) Significantly changed lncRNAs (fold change (L/S) > 2).

It was shown that miRNA expression profiles were significantly different between large and small leiomyomas and there were race and tumor size-specific miRNA signatures in human uterine leiomyomas[5]. To identify mRNA signatures in uterine leiomyomas, we designated large tumor specific mRNAs as set 1 which comprises mRNAs dysregulated only in large tumors. Similarly, we designated tumor size correlated mRNAs as set 2 which comprises mRNAs dysregulated more dramatically in large tumors than in small ones. Then hierarchical clustering and KEGG pathway enrichment analysis were performed in set 1 and set 2, respectively (Figure 3A and 3B). There were nine enriched pathways in set 1 and three enriched pathways in set 2. Importantly, cytokine-cytokine receptor interaction pathway and chemokine signaling pathway are both down-regulated in the two sets (Figure 3A and 3B), which indicates that their down-regulation might contribute to tumorigenesis and progression of leiomyomas.

To elucidate the lncRNA signature for large leiomyomas, we designated large tumor specific lncRNAs as set 3 and tumor size correlated lncRNAs as set 4. We then performed hierarchically clustering in set 3 and set 4, respectively (Figure 3C and 3D). To identify the biological significance of the dysregulated lncRNAs in large tumors, we constructed a 988×949 correlation matrix, including correlation coefficients between mRNAs and lncRNAs (Figure 2B). We next filtered the most credible correlation coefficients based on a cut-off of absolute R > 0.8 and used this matrix to calculate the sum of absolute correlation coefficients with the given lncRNA set for each gene. Then we ordered the genes by the sum of absolute correlation coefficients and selected the genes on the top, the relevant genes that are correlated with two sets of lncRNAs (set 3 and set 4, Figure 3C and 3D) for pathway analysis. 111 relevant mRNAs were down-regulated in large leiomyomas and were enriched in epithelial cell signaling in *Helicobacter pylori* infection and cytokine-cytokine receptor interaction pathway (Figure 3C). Importantly, these two pathways are also down-regulated in set 1 mRNAs that are analysed without consideration of lncRNAs. These results indicate that the downregulation of the two pathways may contribute to the formation of large tumors. Down-regulation of PPAR signaling pathway and up-regulation of N-Glycan biosynthesis pathway were associated with 362 lncRNAs up-regulated in large leiomyomas (Figure 3C). We also analyzed the pathway enrichment in lncRNA set in which lncRNAs were changed more dramatically in large leiomyomas (Figure 3D). Only one pathway, primary bile acid biosynthesis pathway, was found to be significantly enriched. Figure 3E and 3F showed portions of the most dramatically dysregulated mRNAs and lncRNAs, respectively. These results suggest that like dysregulated mRNAs, dysregulated lncRNAs may also serve as signatures of large leiomyomas. With the same approach, we also analyzed mRNAs and lncRNAs in small leiomyomas and found p53 signaling pathway was down-regulated whereas fatty acid metabolism pathway was up-regulated (Supplementary Figure 1).

### Gene Set Enrichment Analysis of three independent expression profile datasets generated possible biomarkers of leiomyomas

**Fig. 4:**
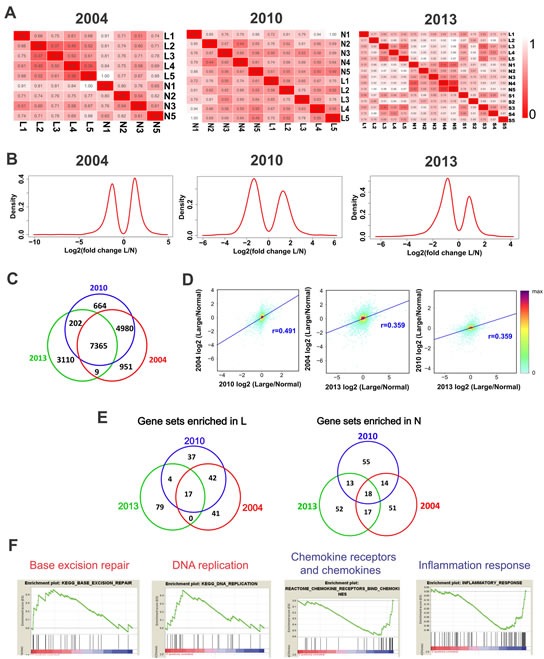
Integrated analysis of three independent datasets of mRNA expression profiles in ULMs (A) The relative Euclidean distance matrix of samples in three independent LMS array datasets. The Euclidean distance between two samples was divided by the maximum distance of two samples in the dataset. (B) The log2 (fold change L/N) distributions of the altered protein-coding genes (fold change >2) in the three datasets. (C) Overlapping of protein-coding genes detected in the three datasets. (D) The log2 (fold change L/N) values were calculated in the 7, 365 common detected genes to derive the Pearson correlations between each two of the three datasets. The pairing density scatter plots of each two datasets were drawn and the Pearson correlations were added. (E) Venn diagrams showing common altered gene sets in the three datasets. (F) The selected enrichment plots of the common altered gene sets. Red and blue colors in gene sets indicate enrichment in L and N respectively.

To determine the validity and reproducibility of the gene expression dataset we obtained, we compared our dataset with two published ones [2, 22]. We constructed relative euclidean distance matrix from three datasets. Homogeneous samples should have shorter distances in the matrix. Judging from this criterion, it appears that the intragroup variations are smaller in 2004 and 2013 datasets than in the 2010 dataset (Figure 4A). Next, we compared the distributions of the dysregulated (log2 fold change >1 or <-1) genes in the three datasets. The distribution plots of the three datasets were similar, especially between the 2010 and 2013 datasets (Figure 4B).

There are totally 7,365 detectable genes in the three datasets (Figure 4C). We calculated the correlation coefficients between datasets by using the log2 fold changes of these 7,365 genes. We found that the 2004 and 2010 datasets are more closely related (r = 0.491) to each other and are more closely related with large leiomyomas samples in our dataset (Figure 4D and S2B). This finding is not surprising considering that the 2004 and 2010 datasets were obtained from large leiomyomas. Gene Set Enrichment Analysis (GSEA) showed that 17 common GSEA gene sets in the top 100 GSEA gene sets are enriched in leiomyomas in all three datasets, and 18 common GSEA gene sets in the top 100 GSEA gene sets are enriched in normal tissues (Figure 4E). Interestingly, base excision repair and DNA replication gene sets were among the enriched in leiomyomas, indicating increased capacities in DNA repair and DNA replication in leiomyomas (Figure 4F). Importantly, chemokine receptors and chemokines gene set and inflammation response gene set are among the 18 common gene sets enriched in normal tissues (Figure 4F), which indicates that inflammation is down-regulated in leiomyomas. This information is consistent with our pathway analysis results in Figure 3.

### Validation of the microarray data using qPCR

**Fig. 5:**
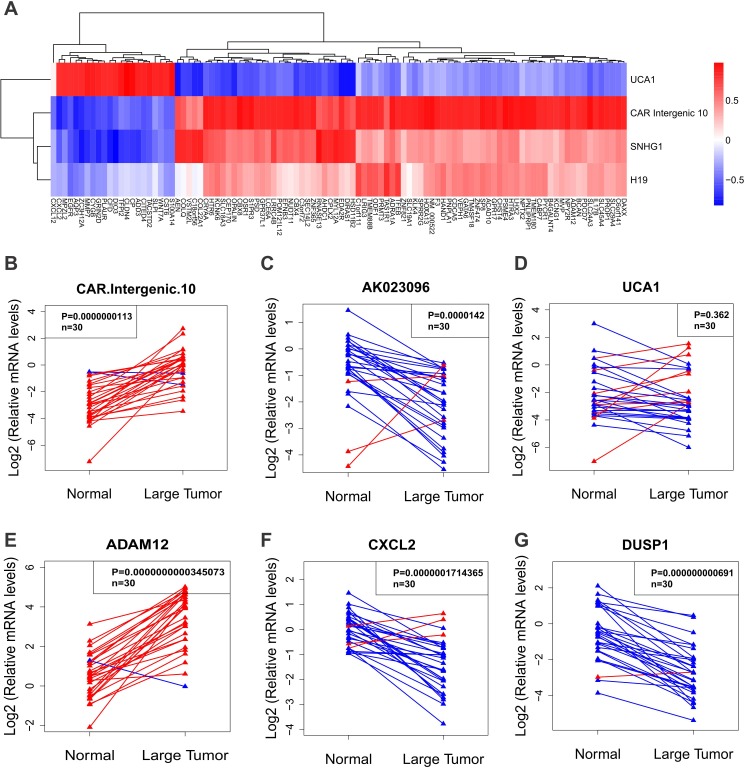
Validation of lncRNA and mRNA expression in uterine leiomyomas compared to normal tissues (A) The correlation matrix of the leading edge genes in the common altered gene sets and significantly changed lncRNAs included in lncRNAdb (http://www.lncrnadb.org/). Red indicates positive correlation, blue negative correlation and white no correlation. (B-D) showed the expression of selected lncRNAs and (E-G) showed the expression of selected mRNAs. Both lncRNAs and mRNAs were measured by qPCR in uterine leiomyoma tissues or adjacent normal tissues (n = 30). Paired t-test was used for comparisons between two groups.

We next validated the credibility of correlation matrix-based integrative approach by determining the levels of selected lncRNAs and the related protein coding genes by qPCR in 30 pairs of matched samples. We chose significantly dysregulated lncRNAs (*CAR Intergenic 10*, *UCA1*, *AK023096*, *BX640708*) and their related protein coding genes (*ADAM12*, *CXCL2*, *DUSP1*, *NEDD9*, *IGFBP6*) in correlation matrix (Figure 2B). CAR *Intergenic 10* was reported to regulate the expression of neighboring genes by establishing active chromatin structures[23]. In our microarray result, *Intergenic 10* was dramatically upregulated in ULMs, with a 9-fold enrichment in small tumors and a 15-fold enrichment in large tumors. qPCR result confirmed the upregulation of *Intergenic 10* in ULMs. It was more highly expressed in leiomyomas than in normal tissues in 28 of 30 cases (Figure 5B). *AK023096* was one of top down-regulated lncRNAs in large leiomyomas, as shown in hierarchically clustered heatmap (Figure 3F). qPCR result showed that *AK023096* had a lower expression level in leiomyomas than in normal tissues in 27 of 30 cases (Figure 5C). *UCA1* and *BX640708* were also shown to be down-regulated in microarray result. However, qPCR result showed that *UCA1* and *BX640708* were down-regulated in only about 70% of 30 leiomyomas compared to normal tissues (Figure 5D, Supplementary Figure 3A). As a protein coding gene, *ADAM12* overlaps with *Intergenic 10* for a few hundred base pairs. Consistent with the microarray result, the expression level of *ADAM12* determined by qPCR was higher in leiomyomas (Figure 5E). *CXCL2* was negatively correlated with *Intergenic 10* based on our integrated analysis (Figure 5A). qPCR result showed that *CXCL2* was down-regulated in the majority of leiomyomas (Figure 5F), which was negatively correlated with *Intergenic 10*. The correlation between I*ntergenic 10* and *CXCL2* expression reached statistical significance (p=0.028; Supplementary Figure 5). We also determined the mRNA levels of several other protein coding genes from lncRNAs and cis mRNAs co-expression network (Supplementary Figure 4). As shown in Figure 5G and Supplementary Figure 3, *DUSP1*, *IGFBP6* and *NEDD9* were down-regulated in the majority of leiomyomas compare to matched normal tissues in accordance with microarray data. Taken together, measurements of lncRNAs and mRNAs in extended samples validated the credibility of our correlation matrix-based integrative approach.

### Functional analysis of CAR *Intergenic 10*

**Fig. 6:**
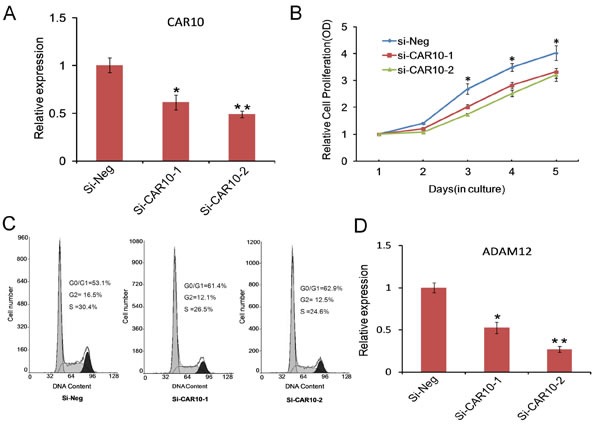
Functional analysis of CAR *Intergenic 10* in ULMs (A) Relative expression of *Intergenic 10* measured by qPCR in DD-HLM cells tranfected with two different CAR Intergenic 10 siRNA sequences or control siRNA. (B) Growth curves illustrated the relative cell proliferation in DD-HLM cells tranfected with *Intergenic 10* siRNAs or control siRNA. (C) Cell cycle distribution was analyzed in DD-HLM cells transfected with with *Intergenic 10* siRNAs or control siRNA. (D) Downregulation of *ADAM12* in *Intergenic 10* knockdown cells.

We next tested whether the dysregulated lncRNAs play any functional roles in ULM development. For this purpose we chose to test *Intergenic 10*, which was remarkably upregulated in ULMs, with a 9-fold enrichment in small tumors and 15-fold enrichment in large tumors. *Intergenic 10* is located on chromosome 10, flanked by *FANK1* (fibronectin type III and ankyrin repeat domains 1) and *ADAM12* (ADAM metallopeptidase domain 12) genes, with an overlap of a few hundred base pairs with *ADAM12* [23]. Expression of *Intergenic 10* was highly related with many leading edge genes of dysregulated GSEA pathways (Figure 5A). We knocked down *Intergenic 10* in leiomyoma cells by using small interfering RNA (siRNA) that was described previously [23]. Interestingly, growth curve data showed that knocking down of *Intergenic 10* with each of two different siRNAs significantly inhibited the proliferation of leiomyoma cells (Figure 6A and 6B). To confirm the growth-inhibitory effect of *Intergenic 10*, we examined the distribution of cell cycles by flow cytometry when *Intergenic 10* was knocked down. As shown in Figure 6C, knockdown of *Intergenic 10* resulted in an increase in the percentage of cells in G0/G1 phase and a decrease in the percentage of cells in S and G2/M phases, indicating *Intergenic 10* induces G0/G1 cell cycle arrest in leiomyoma cells. Taken together, our data suggest that *Intergenic 10* may contribute to the growth of leiomyoma. Moreover, the expression level of *ADAM12* was reduced in *Intergenic 10* knockdown leiomyoma cells (Figure 6D), suggesting that *Intergenic 10* positively regulates the expression of its neighboring gene *ADAM12*.

## DISCUSSION

Previous expression profiling studies of mRNAs and miRNAs in uterine leiomyomas identified many aberrantly expressed protein coding genes and miRNAs and implicated several of them in the pathogenesis of uterine leiomyomas[3, 7]. In this study, we performed expression profiling analysis of lncRNAs as well as mRNAs in uterine leiomyomas to evaluate the genome-wide expression of lncRNAs and mRNAs and their potential role in the pathogenesis of uterine leiomyomas. As lncRNAs were dysregulated in various types of cancers[24-28], we found that hundreds of lncRNAs and mRNAs were differentially expressed in large and small leiomyomas compared to myometrium. There are more aberrantly expressed lncRNAs and mRNAs in large tumors than in small tumors, indicating that large tumors acquire more alterations in gene expression than small tumors. Large leiomyomas are of major clinical concern because patients often seek surgical intervention. It is unclear why only certain uterine leiomyomas progress to become large while tumors others remain small, even in the presence of similar sex hormone status[29]. It is therefore important to identify the pathways and factors that contribute to the tumor growth in larger tumors. We observed that the cytokine-cytokine receptor interaction pathway and chemokine signaling pathway were both down-regulated in large tumors. Furthermore, we developed a correlation matrix-based integrative approach to analyze the lncRNA and mRNA co-expression data and found that the down-regulated cytokine-cytokine receptor interaction pathway was associated with aberrantly dysregulated lncRNAs in large leiomyomas. Fatty acid metabolism pathway, which was up-regulated in small leiomyomas, was also positively correlated with lncRNAs. This finding has clinical relevance since obesity is one of the important risk factors for uterine leiomyomas. Increase in body mass index elevates the risk of developing leiomyomas 2 to 3 times in women[30]. The matrix of correlation coefficients between mRNAs and lncRNAs could help to identify potential functional lncRNAs for further investigation.

Based on our integrated analysis approach and qPCR validation, we found that CAR *Intergenic 10* and its near protein coding gene *ADAM12* were both up-regulated in uterine leiomyomas, which is consistent with previously reported in human fibroblast cells [23]. *CXCL2* was found to be negatively correlated with *Intergenic 10* in co-expression network and this negative association was confirmed by qPCR in extended samples of ULM. Importantly, knockdown of *Intergenic 10* inhibited the proliferation of leiomyoma cells, suggesting that up-regulation of *Intergenic 10* may contribute to the development of ULM. As a dual-specificity phosphoprotein phosphatase *DUSP1* specifically inactivates MAPK signaling related to cell cycle progression and cellular proliferation [31]. Interestingly, *DUSP1* was one of the protein coding genes that are highly correlated with lncRNAs in co-expression network. *DUSP1* was down-regulated in leiomyomas in this study as well as in other studies [16, 31]. It is plausible that MAPK signaling may be activated in leiomyomas because of the decreased inhibition by *DUSP1*, and lncRNAs might be involved in the regulation of MAPK signaling through *DUSP1*.

In summary, we showed for the first time that lncRNAs are aberrantly expressed in uterine leiomyomas compared to myometrium. By applying an integrative approach for the analysis of lncRNAs and mRNAs we identified several pathways that are dysregulated in ULM and are possibly regulated by lncRNAs. Cytokine-cytokine receptor interaction pathway was down-regulated in large leiomyomas, whereas fatty acid metabolism pathway was up-regulated in small leiomyoma. Notably, up-regulation of *Intergenic 10* may contribute to development of uterine leiomyomas. Our integrative analysis of lncRNAs and mRNAs provides a comprehensive insight into pathogenesis of ULMs. It may also be applied to studies that address the functions and mechanisms of lncRNAs in other diseases or conditions.

## MATERIALS AND METHODS

### Patients and tissue samples

Uterine leiomyomas tissues were collected from patients who underwent hysterectomy at the Qilu Hospital, Shandong University between 2011 and 2013. Use of clinical samples in this study was approved by institutional research ethics committee. This study included uterine leiomyomas of usual type from 35 patients. Matched myometrium sample was also collected for each tumor. Tumors were divided into small (≤ 3cm in diameter) and large tumors (≥ 10cm in diameter) according to a previous report [5]. The detail information about patient age, tumor size, number of tumors, was summarized in Supplementary Table 1.

### Tissue preparation and RNA extraction

Tissue samples were collected within 2 hours of surgery. All fresh tissues were sliced to 5mm^3^ in size and immersed in 10 volumes of RNALater (Ambion, Austin, TX). The tissue samples were then stored in a -80°C laboratory freezer. RNA was isolated using TRIzol reagent (Invitrogen) according to the manufacturer's protocol.

### Microarray and expression data sets

For microarray analysis, five groups of matched samples were used, including five myometrium samples, five small tumor samples and five large tumor samples. RNA was purified from total RNA after removal of rRNA (mRNA-ONLY™ Eukaryotic mRNA Isolation Kit, Epicentre). Then, each sample was transcribed into fluorescent cRNA along the entire length of the transcripts without 3' bias utilizing a random priming method. The labeled cRNAs were hybridized onto the Human lncRNA Array v2.0 (8 x 60K, Arraystar). The arrays were scanned by the Agilent Scanner G2505B. Agilent Feature Extraction software (version 11.0.1.1) was used to analyze acquired array images. Quantile normalization and subsequent data processing were performed using the GeneSpring GX v11.5.1 software package (Agilent Technologies). The microarray data generated in this study have been deposited in NCBI GEO database under the accession number GSE52618. The mRNA expression profile data sets from uterine leiomyomas previously published by Hoffman *et al* (GSE593)[22] and Zavadil *et al* (GSE23112)[2] were obtained from the NCBI GEO database.

### Bioinformatics analyses

R-3.0.1 (http://www.r-project.org/) was utilized to calculate the Pearson correlations between mRNAs and lncRNAs and to generate density distributions. For the pathway enrichment analysis, DAVID Bioinformatics Resources 6.7 (http://david.abcc.ncifcrf.gov/ ) was utilized. Gene Set Enrichment Analysis (GSEA) was performed with GSEA software (http://www.broadinstitute.org/gsea/index.jsp). Gene sets were exported from MSigDB using CP (Canonical pathways).

### Quantitative Real-Time PCR

Total RNA was isolated using the TRIzol(Invitrogen), and cDNA was synthesized with RevertAid™ M-MuLV Reverse Transcriptase (Fermentas) using random hexamer primers according to manufacturer's instruction. SYBR Green real-time PCR (Applied Biosystems) was used to detect the expression of lncRNAs and mRNAs. Small nuclear RNA U6 was used as an internal control. Primers used in this study are summarized in Supplementary Table 2.

### Cell culture and siRNA transfection

Human immortalized leiomyoma cell line DD-HLM cells were obtained from Dr Ayman Al-Hendy (Meharry). The cell line has been tested and authenticated as described previously [32]. Cells were maintained in DMEM/F12 (1:1) (Invitrogen) medium with 10% fetal bovine serum (USA Scientific). The siRNA oligonucleotides designed for CAR *Intergenic10* were synthesized (GenePharma, Inc. China) as described previously[23]. siRNA (50 nM) was transfected using Lipofectamine 2000 (Invitrogen) according to the manufacturer's instructions. 48 hours after transfection, cells were harvested and analyzed for knockdown efficiency. Growth curve and cell cycle analysis were used to evaluate the effect of *Intergenic 10* knockdown on cell proliferation.

### Cellular proliferation assay

Cellular proliferation assay was performed as described previously[33]. Briefly, the WST-1 (Roche) was used for cell proliferation assay according to the manufacturer's instructions. Cell proliferation was monitored at different times (1–5 days).

### Cell cycle analysis by flow cytometry

Cell cycle analysis was performed as described previously[34]. The transfected cells (24 hour post-transfection) were plated at a concerntration of 5×10^4^ cells/ml in 60 mm dishes and incubated for further 48 h. The cells were fixed and stained with 50μg/ml propidium iodide. Cell cycle distribution was analyzed using a flow cytometer(Beckman Coulter, Inc.).

### Statistical analysis

All data are presented as means and standard errors in triplicate. Student's t-test was used for comparisons between two groups of experiments, and one-way ANOVA was used for comparisons among three or more groups. Statistically significance was considered as: ^*^P≤ 0.05 or ^**^P≤0.01.

## SUPPLEMENTARY MATERIAL, FIGURES AND TABLES


