# Supplementary material for: Integrated analysis of long noncoding RNAs and mRNAs reveals their potential roles in the pathogenesis of uterine leiomyomas
Source: Oncotarget. 2014 Aug 16;5(18):8625–36. doi: 10.18632/oncotarget.2349 (PMC4226709; doi:10.18632/oncotarget.2349)
Supplement: Supplementary file 1 [file oncotarget-05-8625-s001.pdf]

Integrated analysis of long noncoding RNAs and mRNAs reveals their potential roles in the pathogenesis of uterine leiomyomas

Supplementary Material

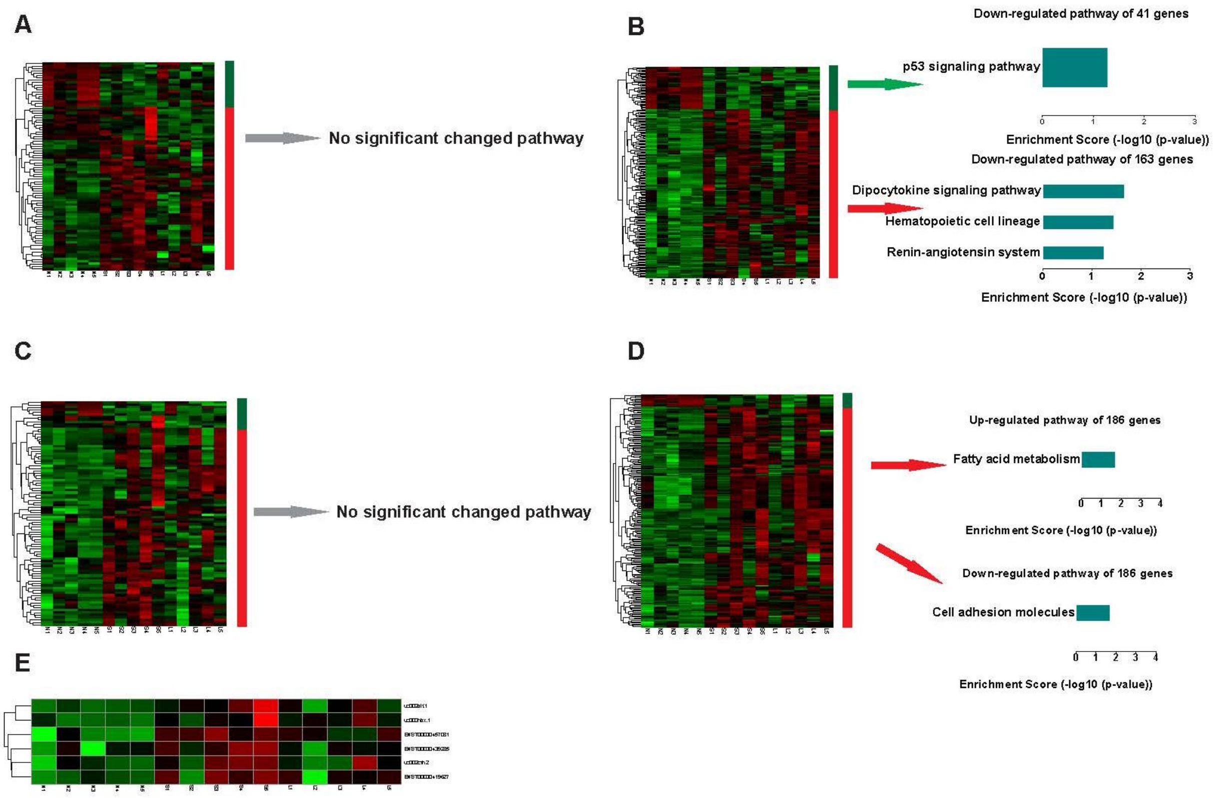

Supplementary Figure 1: Signatures of altered mRNA and lncRNA expressions in small leiomyomas. (A) Left: A hierarchically clustered heatmap of mRNAs changed (Fold change > 2) in small fibroids but not changed in large fibroids. (B) Left: A hierarchically clustered heatmap of mRNAs more dramatically changed in small fibroids than in large fibroids. Right: Significantly enriched pathways of the indicated gene sets. (C) Left: A hierarchically clustered heatmap of lncRNAs changed (Fold change > 2) in small fibroids but not changed in large fibroids. (D) Left: A hierarchically clustered heatmap of mRNAs more dramatically changed in small fibroids than in large fibroids. Right: Significantly enriched pathways of genes correlated with the indicated lncRNA sets. (E) LncRNAs significantly changed (Fold change (S / L) > 2).

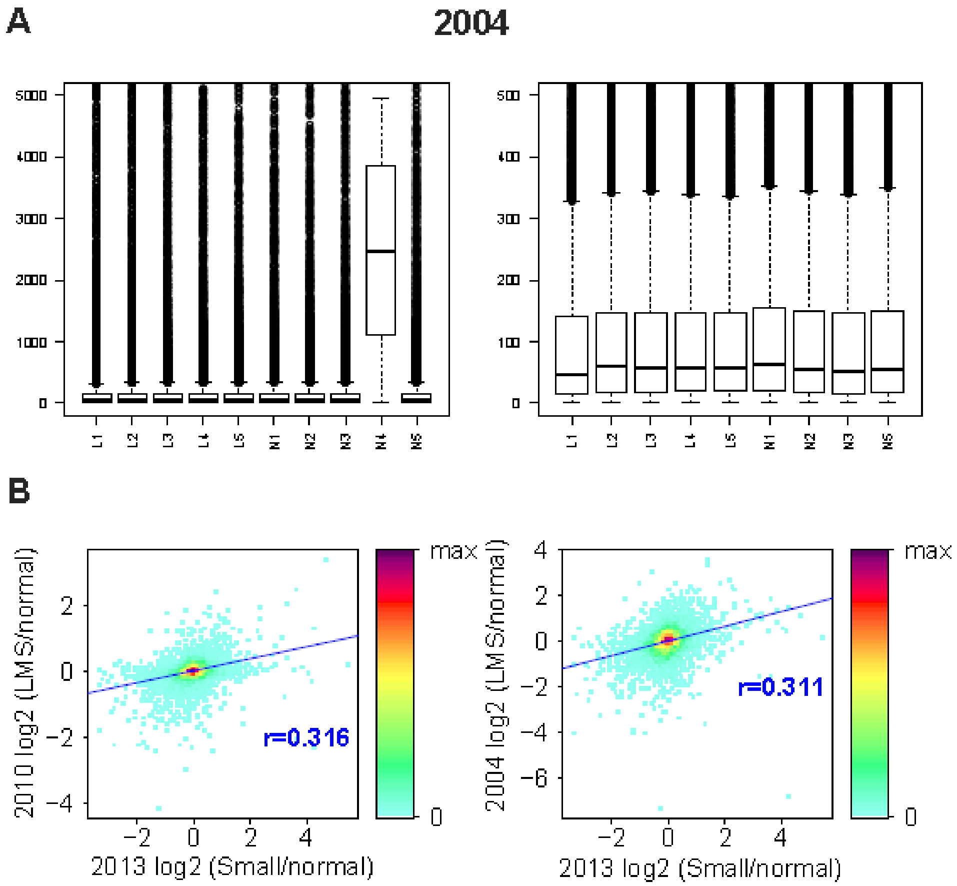

Supplementary Figure 2: Processing of published data and comparison of mRNA expression between our data and published data. (A) The box plots of data set published in 2004. Left: The box plot of the data in soft file downloaded from GDS484 data set in GEO database. Right: The box plot of GDS484 data removing sample N4. (B) The log2 (fold change S/N of 2013 data and fold change LMS/N of 2004 and 2010 data) values were calculated in the 7365 common detected genes to derive the Pearson correlations between each two of the three data sets. The pairing density scatter plots of each two data sets were drawn and the Pearson correlations were added.

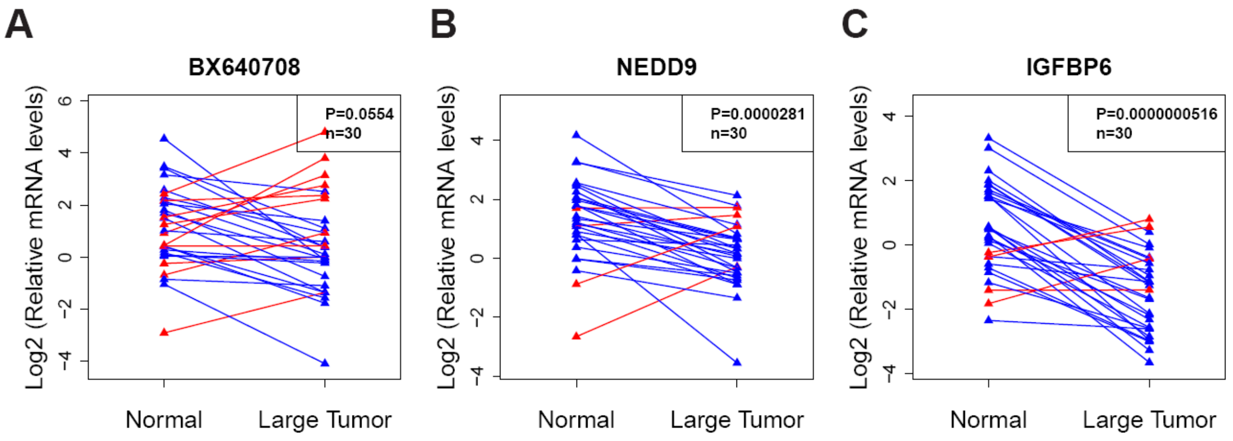

Supplementary Figure 3: Validation of lncRNA and mRNA expression in uterine leiomyoma compared to normal tissues. (A) showed the expression of lncRNA BX640708 and (B,C) showed the expression of NEDD9 and IGFBP6. Both lncRNAs and mRNAs were measured by real time RT-PCR in uterine leiomyomas tissues tissues or adjacent normal tissues (n = 30). Paired t-test was used for comparisons between two groups of experiments.

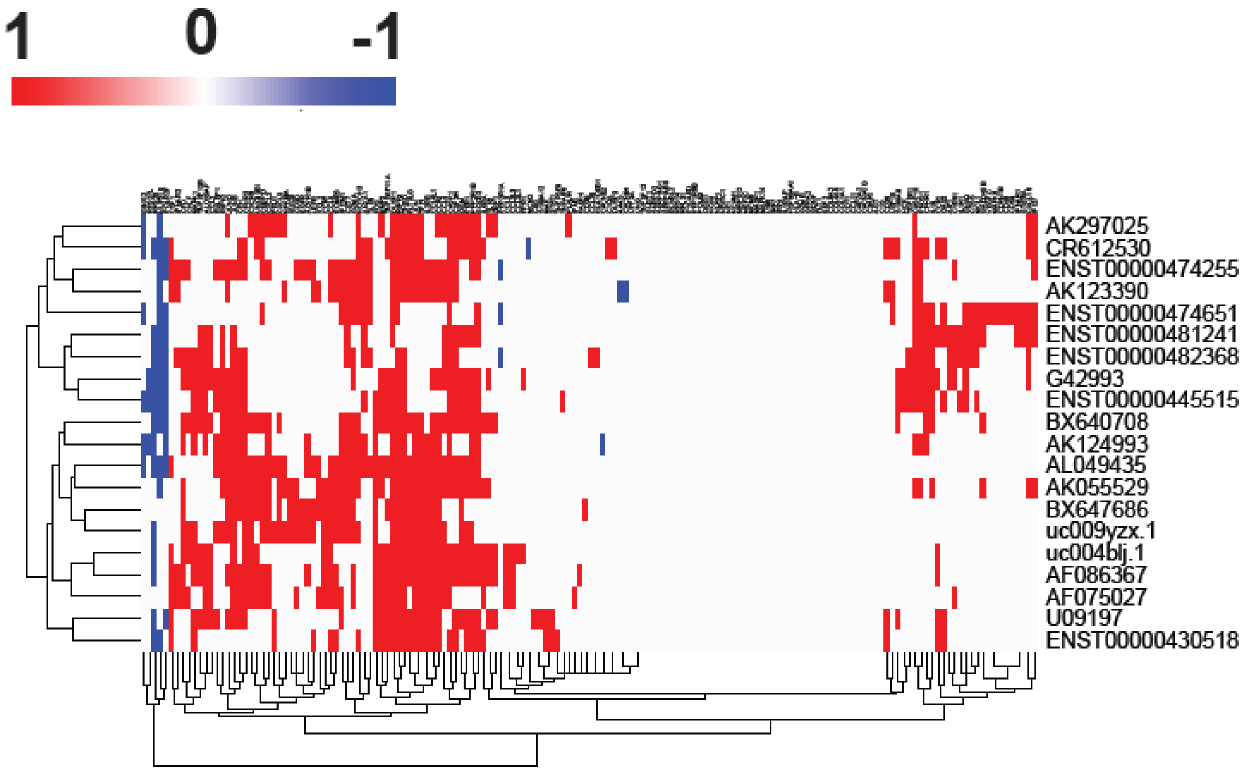

Supplementary Figure 4: The correlation matrix of the leading edge genes in the common altered gene sets and top 20 lncRNAs. Red color indicates positive correlation, blue color means negative correlation and white color means no correlation.

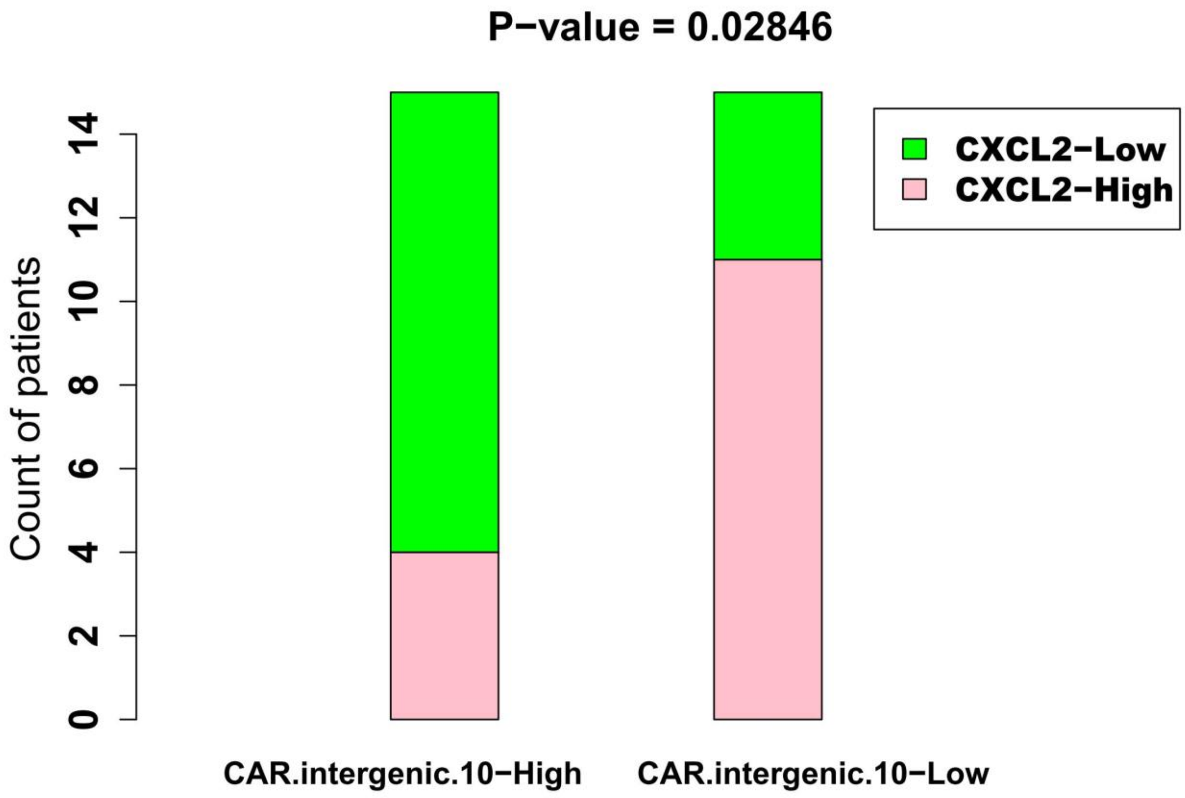

Supplementary Figure 5: Pearson's chi-square test was used to assess the correlation between CAR.intergenic.10 and CXCL2 expression.

Supplementary Table 1: Patients information

Case No.	Age(years)	No. Tumors(n)	Largest tumor size(cm)
ULM1	36	15	14
ULM2	46	6	10
ULM3	53	4	2.5
ULM4	46	3	9.5
ULM5	50	1	3
ULM6	36	⩾10	9
ULM7	49	9	3.5
ULM8	46	⩾3	6
ULM9	43	⩾3	6
ULM10	49	4	5.5
ULM11	50	6	5.5
ULM12	58	1	1.4
ULM13	46	4	3
ULM14	43	3	4
ULM15	49	5	7.5
ULM16	45	5	5
ULM17	48	3	8.5
ULM18	45	11	7.5
ULM19	44	4	2
ULM20	48	2	9.2
ULM21	50	8	8
ULM22	57	5	7

ULM23	49	1	9
ULM24	50	1	15.5
ULM25	50	2	5
ULM26	48	3	2.5
ULM27	44	14	8
ULM28	47	3	6
ULM29	46	⩾10	3.5
ULM30	51	5	7
ULM31	48	⩾3	4
ULM32	52	5	2.7
ULM33	50	⩾3	7
ULM34	52	⩾3	9
ULM35	47	⩾10	8.5

Supplementary Table 2: Primers used in this study

Method	Primer Name	Primer sequence(5'—3')
QRT-PCR	CAR Intergenic 10 (F)	TCTGCTGGACTTAGGCTGGT
QRT-PCR	CAR Intergenic 10 (R)	TGCTGCAGTGTGTGGCTATC
QRT-PCR	UCA1(F)	GGGACTCCTTCGTGAGACC
QRT-PCR	UCA1(R)	AGAGGAACGGATGAAGCCTG
QRT-PCR	AK023096 (F)	CGTGGACAAATGCAAGCACA
QRT-PCR	AK023096(R)	GGAAATGGAATGCACGCTGG
QRT-PCR	BX640708 (F)	ATCCCCAACTCTGCAACTGG
QRT-PCR	BX640708(R)	TTAATGGAAGGGCCACCCAC
QRT-PCR	ADAM12(F)	CAGTTTCACGGAAACCCACT
QRT-PCR	ADAM12(R))	AGTCCCCTGAGACCAGAACA
QRT-PCR	CXCL2(F)	TAGCCACACTCAAGAATGGGCAGA
QRT-PCR	CXCL2(R)	ACAGCCACCAATAAGCTTCCTCCT
QRT-PCR	DUSP1(F)	AGTACCCCACTCTACGATCAGG
QRT-PCR	DUSP1(R)	GAAGCGTGATACGCACTGC
QRT-PCR	NEDD9(F)	ATGGCAAGGGCCTTATATGACA
QRT-PCR	NEDD9(R)	TTCTGCTCTATGACGGTCAGG
QRT-PCR	IGFBP6(F)	GAGGGGCTCAAACACTCTACG

QRT-PCR	IGFBP6(R)	CCATCCGATCCACACACCA
QRT-PCR	U6(F)	CTCGCTTCGGCAGCACA
QRT-PCR	U6(R)	AACGCTTCACGAATTTGCGT

Supplementary Table 3: Gene sets enriched in large tumors and normal tissues

**Gene sets enriched in L in all three data sets**	**Gene sets enriched in N in all three data sets**
REACTOME_INFLUENZA_VIRAL_RNA_TRANSCRIPTION AND_REPLICATION	PID_IL12_2PATHWAY
REACTOME_ACTIVATION_OF_THE_PRE_REPLICATIVE_C OMPLEX	REACTOME_COMPLEMENT_CASCADE
KEGG_DNA_REPLICATION	KEGG_COMPLEMENT_AND_COAGULA TION_CASCADES
REACTOME_NONSENSE_MEDIATED_DECAY_ENHANCED _BY _THE_EXON_JUNCTION_COMPLEX	REACTOME_CHEMOKINE_RECEPTORS _BIND_CHEMOKINES
REACTOME_PEPTIDE_CHAIN_ELONGATION	KEGG_NOD_LIKE_RECEPTOR_SIGNALI NG_PATHWAY
REACTOME_ACTIVATION_OF_ATR_IN_RESPONSE_TO _REPLICATION_STRESS	PROTEASE_INHIBITOR_ACTIVITY
KEGG_RIBOSOME	KEGG_CYTOKINE_CYTOKINE_RECEPT OR_INTERACTION
REACTOME_NCAM1_INTERACTIONS	REACTOME_INNATE_IMMUNE_SYSTE M
MIPS_RIBOSOME_CYTOPLASMIC	RESPONSE_TO_WOUNDING
KEGG_BASE_EXCISION_REPAIR	PID_AP1_PATHWAY
REACTOME_DNA_STRAND_ELONGATION	INFLAMMATORY_RESPONSE
STRUCTURAL_CONSTITUENT_OF_RIBOSOME	PID_IL6_7PATHWAY
MIPS_60S_RIBOSOMAL_SUBUNIT_CYTOPLASMIC	RESPONSE_TO_EXTERNAL_STIMULUS
MIPS_40S_RIBOSOMAL_SUBUNIT_CYTOPLASMIC	KEGG_DORSO_VENTRAL_AXIS_FORM ATION
REACTOME_EXTENSION_OF_TELOMERES	REACTOME_INTERFERON_ALPHA_BET A_SIGNALING
SMALL_NUCLEAR_RIBONUCLEOPROTEIN_COMPLEX	BLOOD_COAGULATION
REACTOME_INFLUENZA_LIFE_CYCLE	WOUND_HEALING

	COAGULATION
